# Modeling the Formation Process of Grouping Stimuli Sets through Cortical Columns and Microcircuits to Feature Neurons

**DOI:** 10.1155/2013/290358

**Published:** 2013-11-28

**Authors:** Frank Klefenz, Adam Williamson

**Affiliations:** ^1^Division of Bio-Inspired Computing, Fraunhofer IDMT, 98693 Ilmenau, Germany; ^2^Department of Nano-Biosystem Technology, Ilmenau University of Technology, 98693 Ilmenau, Germany

## Abstract

A computational model of a self-structuring neuronal net is presented in which repetitively applied pattern sets induce the formation of cortical columns and microcircuits which decode distinct patterns after a learning phase. In a case study, it is demonstrated how specific neurons in a feature classifier layer become orientation selective if they receive bar patterns of different slopes from an input layer. The input layer is mapped and intertwined by self-evolving neuronal microcircuits to the feature classifier layer. In this topical overview, several models are discussed which indicate that the net formation converges in its functionality to a mathematical transform which maps the input pattern space to a feature representing output space. The self-learning of the mathematical transform is discussed and its implications are interpreted. Model assumptions are deduced which serve as a guide to apply model derived repetitive stimuli pattern sets to *in vitro* cultures of neuron ensembles to condition them to learn and execute a mathematical transform.

## 1. Introduction

It can be said that neuronal networks, whether artificial, *in vivo*, or *in vitro*, are capable of information processing if they are able to learn and discriminate between pattern sets [1–3]. The central focus in modeling the information processing of such networks is on the specific neuronal architecture which is trained. This is because the architecture of the network determines the possible pattern discriminations that can be performed between pattern sets. For example, a specific architecture may provide orientation selectivity and thus be capable of discriminating between bars of different slopes. Bioinspired concepts will be introduced in the first section of this review with emphasis on the aspects of the *in vivo* experiments of orientation selectivity by Hubel [4]. Furthermore, the hypothesis of Blasdel will be revisited in the section on the Hough transform in the neurobiological context. The hypothesis states that the firing of these orientation selective cells can be explained by mapping the input stimuli back to the firing cells using a mathematical Hough transform [5]. To strengthen the plausibility of Blasdel's hypothesis, the motion-detection experiments of Okamoto et al. are also revisited which investigated the hypothesis under the assumption that the mathematical Hough transformation is functionally used and represented as microcircuitry for bar detection in the medial temporal lobe (MTL) of the brain [6].

The base principle of the mathematical Hough transform will be outlined in the section on computational Hough models at the neural level. As the interconnection scheme and interplay of the associated neurons in the microcircuitry of orientation selectivity remain open, we additionally describe with particular attention the modeling and execution of the mathematical Hough transform. Several computational models are contrasted, beginning at the microcircuitry level of the interconnected neurons and synapses. A prime example of this, namely, a neural net composed of cortical columns and microcircuits, is discussed in detail.

Following these sections, we conclude by proposing guidelines and novel 3D microelectrode arrays (MEAs) to be used in future *in vivo* bar detection experiments with stem cell derived neuron-glia ensembles *in vitro*. They will be stimulated according to a new protocol presented here with spatiotemporal bar patterns. The proper selection of stimuli pattern sets is explained. The methods for stimulation and the experimental setups are described in detail.

## 2. Overview of Bioinspired Concepts of Orientation Selectivity

The sensory organs provide the windows to the world for the brain. Eyes and ears encode photon distributions or sound pressure levels into electrical spike trains which are delivered and distributed by *cranial nerves* and optic nerves to higher cortical information processing areas [7, 8]. Many sensory stimulation experiments have been conducted in the last few decades to reveal basic principles of information processing in the brain [9, 10]. The *in vivo* work of these experiments has primarily been conducted with anaesthetized animals which are then presented with visual or auditory stimuli [11]. The visual system is often stimulated with specific visual cues such as moving bars or natural videos and the auditory system with preconditioned sounds, natural speech, and music [12]. *In vivo* experiments presumably clarify the encoding and information processing principles of the brain more accurately than *in vitro* experiments with cultures [13–15].

The sensory organs mediate the information transfer from the outer world to the inner world. This mediated information in the inner world is used to create internal representations of the outer world in distinct brain areas and is structured in the brain into categories, such as color and motion in the visual system [16]. In a famous experiment, Hubel and Wiesel presented bars of different orientations to the visual receptive fields of cats [17]. They found that orientation selective neurons respond most to visual stimuli like bars of different slopes presented in their receptive field in their preferred orientations. Orientation specific cells fire more strongly as the presented image bars are oriented more towards the cell's preferred orientation. For example, preferred orientation would be horizontal orientation for one cell and vertical orientation for another cell. The firing rate declines if the presented image bar is more and more off-line in reference to the preferred direction. Their experiments revealed that specific orientation selective cells are to be considered as bar detectors. However, the underlying cellular micro-circuitry from the retina to the orientation selective cells was not discussed in detail. Many models to understand the organization of this micro-circuitry which yields orientation selectivity have been proposed, listed here in chronological order [18–21]. In the following section, we detail the most successful model which utilizes the Hough transform.

## 3. The Hough Transform in a Neurobiological Context

The hypothesis that the firing of orientation selective cells could be understood by mapping their input stimuli by a mathematical transform which is intrinsically implemented in cellular microcircuits was formulated by Blasdel. He demonstrated that a mathematical Hough transform model could be adopted to explain principles of the transformational process of bar detection by orientation selective cells. The Hough transform is a coordinate transform, in which an input space is transformed to an ordered feature space. Each point in the feature space is given by its coordinates and numerically by the accumulation vote of histogram entries in the corresponding grid cells. Blasdel assumes a coordinate transform mapping of the (*x*, *y*) input pixel space of lines to a feature space which is topologically spanned by ordering polar coordinates (*r*, *θ*) of radii and angles of lines. Any extended line composed of several pixels will be represented in feature space solely by a single point representation given by its specific radius and angle [22]. An example of this is the parallel execution of the Hough transform on a matrix of numerical grid elements to find curved tracks in drift chambers for high-energy physics experiments and its corresponding hardware implementation [23, 24]. The highly structured Hough feature space with ascending and descending orientation angles *θ* in one axis and ascending and descending radii *r* in the other makes it a compelling model for orientation mapping in the striate cortex.

Indeed, Kawakami and Okamoto propose a cell model for motion detection in which the Hough transform is the essential part in the identification of bars [25]. They compare it to several propositions from the literature and conclude with a model consisting of five cell types which constitute a functional hierarchy for motion detection: lateral geniculate nucleus (LGN) cells, nondirectionally selective (NDS) simple cells, directionally selective (DS) simple cells, DS complex cells, and motion-detection cells. Their motion-detection model and the consecutive processing steps start with lagged and nonlagged branches in the magnocellular visual pathway which split the actual representations in a delayed and nondelayed version. The local motion is detected as the spatial distance between the identified object primitives in the delayed and nondelayed images. Object primitives like bars are detected in the MTL with a Hough transform collectively executed by NDS cells. Motion is estimated in the MTL consecutively by a spatiotemporal correlation and an inverse Hough transform. It is a coherent description of a chain of processing units in coordinate spaces by a structured assembly of cells.

Equipped with this model and model predictions, Okamoto et al. conducted a motion-detection experiment with primates. They extensively compared their model predictions to *in vivo* measurement of macaque monkeys presented with moving shapes of different velocities. They conclude that for the estimation of the speed of moving bars and of moving spots MTL neurons exhibited two types of bimodal direction tuning profiles as predicted by their model. This experiment demonstrated that a functional *in vivo* system can be successfully represented by a mathematical model, and implies that the essential algorithm in Hough bar detection is incorporated at least partially in the MTL.

## 4. Computational Hough Models at the Neuron Level

Although successful in its predictions, Kawakami's model remains a mathematical model with little indication of how the algorithm is realized cellularly. In what way this algorithm might be executed structurally, dynamically, and functionally by a neuron ensemble remains largely unknown. The base model descriptors are neurons as entities which are affected with some model assumptions [26]. Most neurons are modeled as integrate and fire devices, which propagate an action potential along their axon, if a threshold is passed at its soma [27]. The neuron is excited by the sum of synaptic inputs which triggers an action potential if the net input surpasses the activation threshold.

Several authors describe spiking neural network models which execute Hough transforms at the neuron and synapse level [28–31]. Their implementation aspects, architectures, topologies, and learning dynamics have been compared. The computational aspect of how the orientation cells are coupled in their receptive fields to the sensory layers and the functional dynamics of algorithmic computation by an ensemble of neurons in these microcircuits is discussed. All four authors selected for this synopsis present feedforward artificial neural networks (ANNs) with unidirectional signal flow without any feedback loops. Feedforward ANNs are used extensively in pattern recognition for eye tracking and pencil balancing [32, 33].

In the first Hough transform implementation, the authors describe a character recognition study using a biologically plausible neural network of the mammalian visual system [28]. Vertical and horizontal line elements are extracted by a Hough transform. The feedforward ANN incorporates horizontal and vertical line detectors with five layers: input units, simple line detectors, complex line detectors, hidden units, and output units. The connectivity is taken to be a many-to-many mapping. Each input neuron is associated with a synaptic link to a hidden layer neuron and each hidden layer neuron is connected to an output classifier neuron. The network is trained by element training sets composed of vertical and horizontal line elements. The training elements are presented over and over to the artificial neural network in a pixel grid of size 5 × 7. To recognize a given element, the connecting weights adapt for error minimization between the desired output and the actual output. A back-propagation learning algorithm is used to set the synaptic weights from the input layer to the hidden layers and the weights from hidden layers to the output layer. The character recognition performance and the efficiency of the neural network using line detectors in the early layers are superior to that of a network using adjustable weights. The opinion of the authors is that the system should be extended to four line categories: vertical, horizontal, ascending diagonal, and descending diagonal.

In their second implementation of the Hough transform, the authors further described a biologically inspired spiking neural network with Hebbian learning for vision processing. The authors wrote that the Hough transform can be used to find simple shapes like lines and circles in images [29]. Their network input layer consists of 9 input neurons in a 3 × 3 pixel array. These input neurons are connected to one output neuron. The net was trained on two input patterns: a horizontal bar and a vertical bar with a Hebbian-style unsupervised learning rule. The authors described that the spiking neutral network is able to learn and discriminate between the patterns.

In their third implementation, the authors demonstrated the detection of straight lines using the above described spiking neural network model. Based on the receptive field of the Hough transform, the authors showed that a spiking neural network is able to detect straight lines in a visual image [30]. Straight lines on the plan can be identified by a pair of polar coordinates (*r*, *θ*), where *r* is the perpendicular distance from the line to a reference point at the origin (0,0) and *θ* is the angle perpendicular to the line and the horizontal axis. A specific line detector neuron is associated with each line. The line classification neurons are arranged in an array with the two ordering parameters *r* and *θ*. In this spiking neural network model, each neuron in the output array responds to a specific line in a visual image. Each of the classifier neurons is connected by excitatory synapses to the constituting pixel sets of a line. The authors write that the spiking neutral network is able to learn and discriminate between the lines. In the outlook of the publication, they pose the following questions: how can the model exist in the biological visual system and how should a spike timing dependent plasticity (STDP) rule for synapses be investigated?

**Figure 1:**
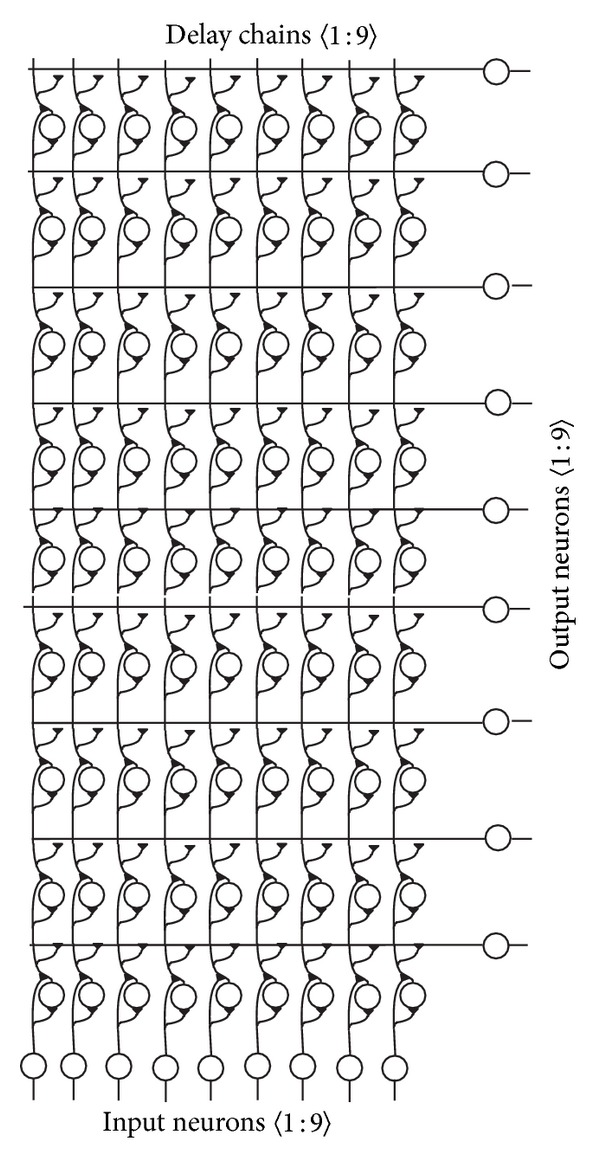
A feedforward neural network model with an input layer at the bottom and feature classifier output neurons at the right. The network topology is regular with repetitive elementary building block segment microcircuits. (Reprinted with permission from Brückmann et al., [31].)

In their fourth implementation, the authors described a neural net for 2D slope and sinusoidal shape detection [31]. Again, based on the receptive field of the Hough transform, the authors discussed a spiking neural network. However, this time it was demonstrated that with the cortical column architecture it is possible to detect straight lines or sinusoids in a visual image according to the presented training sets. The neural net consists of *m* input neurons, *m* × *n* delay neurons, and *n* output neurons (Figure 1). The net is structured by column architecture with concatenated chains of delay neuron as building blocks. The input neuron layer is a first row of neurons which receive spatiotemporal signal input in parallel. An output column composed of feature classifier neurons is displayed on the right side (Figure 1). The classifier neurons are equidistantly interspaced and perpendicular to the parallel cortical columns. The output neurons are linearly aligned with ascending slopes from slope 0 which corresponds to a horizontal bar (feature neuron at the bottom of the column) up to slope 45° which corresponds to a diagonal bar (feature neurons at the top of the column). The classifier neurons are modeled as receiving synaptic inputs at their dendrite from each column related to their topological arrangement in the net [34]. The dendritic branch is modeled according to the base assumptions of dendritic integration of net synaptic input across the soma during a fixed time window [35]. The synaptic interconnection to the dendrite is modeled as a single spine to the dendrite [36]. A dendritic delay along the dendrite is not explicitly modeled as a possible computational function [37].

The neural net learns to detect a set of training elements like bars or sinusoids. It is trained with a set of *k* different bars or *l* sinusoids of different frequencies. The training and test patterns are 2D binary pixel images of size *m* × *n*. A typical set of bar training patterns of image size 9 × 9 is displayed in Figure 3. Each consecutive new time step for each subsequent image row is applied at the corresponding input layer in a spatio-parallel, temporally serialized fashion (Figure 3). After training, the net is tested with a random set of 2D bar or sinusoidal patterns and is able to discriminate *n* different patterns.

The spatiotemporal input patterns are transformed to a time and place code where the firing of an output neuron *i* signals the presence of a bar with its specific slope at relative time *t*.

The input layer feeds the subsequent layers with the spatiotemporal input patterns and triggers a propagation of the signals through the associated cortical columns. Each spatiotemporal input, such as a bar with a defined slope, activates the propagation of a wave front in the net through its cortical columns. Due to the specific signal propagation velocities in the net, the activation wave front forms a planar wave front at a specific layer *k* at a specific time *t* for a specific input. The output neuron *k* spikes upon arrival and registration of a planar wave front according to its selective input pattern. The activation wave front rapidly dissolves before or behind layer *k* due to the different signal propagation velocities.

The signal propagation velocity vector field of the cortical columns delay lines is learned by collectively tuning their individual signal propagation velocities. Each cortical column delay line adapts in a pattern induced learning process to its specific signal propagation velocity. The delay times of all delay neurons are equal and set to 1 millisecond (the duration of a clock step).

**Figure 2:**
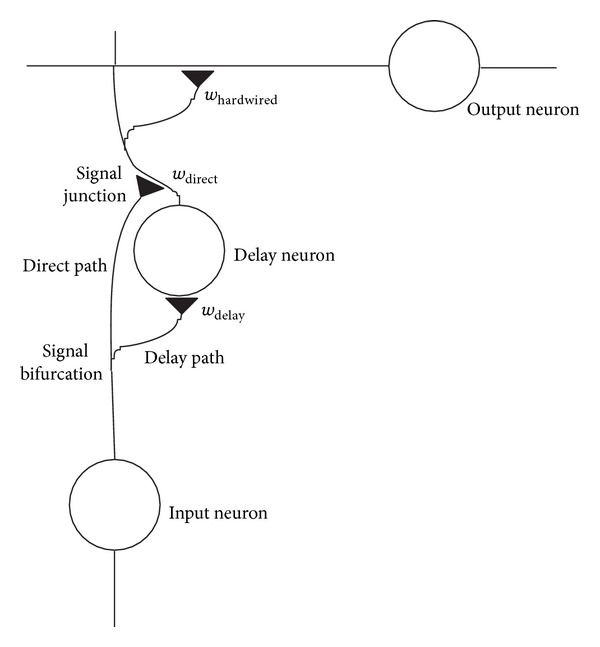
The elementary building block segment microcircuit. Path following is self-learned by the settings of the weights at the signal bifurcation. (Reprinted with permission from Brückmann et al., [31].)

**Figure 3:**
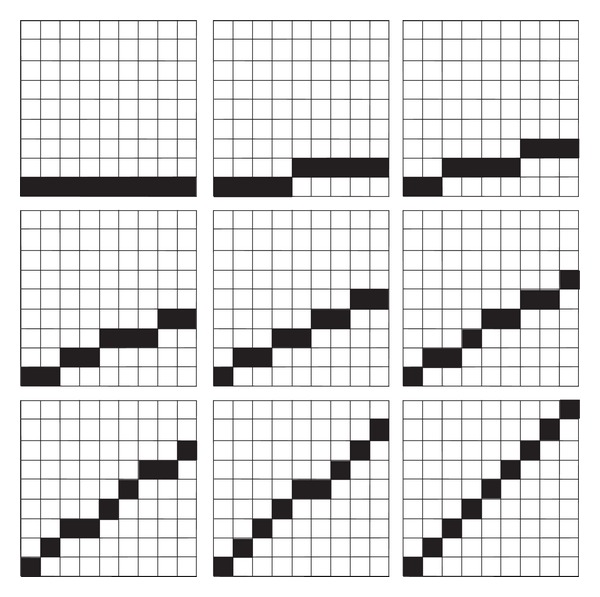
Nine training patterns of bars of different slopes. Each time step one image row is consecutively applied to the input layer. (Reprinted with permission from Brückmann et al., [31].)

Each cortical column delay line consists of a signal conducting pathway (Figure 2). The microscale architecture of a cortical column delay line is given by its composing elements and its specific connectivity. The delay line is composed of an elementary building block segment microcircuit repetitively staggered *n* times. Each micropathway in a block segment branches at a signal path bifurcation into a signal delay path and a direct path. Both paths recombine at a signal junction (Figure 2).

The path selection and therefore the signal propagation local velocity are regulated by antagonistic weights *w*_*ij*,delay_ and *w*_*ij*,direct_. Antagonistic weights *w*_delay_ and *w*_direct_ are shown in Figure 2. By adjusting these weights according to the applied learning rule, the signal propagation velocities in the delay lines are collectively tuned. The weights *w*_*ij*,delay_ and *w*_*ij*,direct_ are in the range [0, 1] and are initially set to 0.5. The input layer differs from the other subsequent layers in that the direct path weights *w*_*ij*,direct_ are set to 1 clamping the paths of the input layer directly to the first output neuron. The synaptic interconnections *w*_*ij*,hardwired_ to the output neurons are hardwired (*w*_*ij*,hardwired_ = 1) (Figure 2). As the weights converge to 0 or 1, they act as gating or closing switches and propagate the signal through or block it.

The synaptic weights are trained with an unsupervised Hebbian-learning rule and a Boltzmann temperature function which decreases from a starting temperature *T*_max⁡_ to a lower end temperature *T*_min⁡_ in constant amounts *δT* [38]. Each layer has its own Boltzmann temperature. The following learning rule applies for all subsequent layers. A random number in the range [0,1] is computed for every signal bifurcation. The probability of the direct or delay signal path being taken at a signal bifurcation is computed by a Boltzmann temperature dependent term as follows:

(1) $$\begin{array}{c} P_{\text{direct}}=\frac{e^{w_{ij,\text{direct}}/T_{\text{Boltz}}}}{e^{w_{ij,\text{direct}}/T_{\text{Boltz}}}+e^{w_{ij,\text{delay}}/T_{\text{Boltz}}}},\\ P_{\text{delay}}=1-P_{\text{direct}}. \end{array}$$

The random value at each node is then compared with the probability of the direct path. If the random number is greater than or equal to *P*_direct_, the delay path is activated. If the random number is less than *P*_direct_, the direct path is activated.

An output neuron only spikes if all selected signal paths are activated, because the thresholds of the output neurons are equally set to the number of signal paths minus 1. The thresholds of the output neurons can be adjusted to lower values (e.g., the output neuron spikes if more than *k* inputs are active). This could accelerate the learning process and be more robust to noise or defective structures, that is, complete loss of several delay lines, and so forth.

If an output neuron spikes in a layer, the weights of the selected paths are collectively changed by +*ε* and the other by −*ε*. *α* is the learning slope parameter, which influences the convergence after *n* iterations to a stable end state. The weights are updated by

(2) $$\begin{array}{c} w_{ij,\text{select},\text{new}}=w_{ij,\text{select},\text{new}}+\left(1-w_{ij,\text{select},\text{old}}\times \alpha \right),\\ w_{ij,\text{deselect},\text{new}}=w_{ij,\text{deselect},\text{new}}-w_{ij,\text{deselect},\text{old}}\times \alpha . \end{array}$$

The two weights *w*_*ij*,delay_ and *w*_*ij*,direct_ in the signal bifurcation paths are always simultaneously changed. The weights *w*_*ij*,delay_ are computed as *w*_*ij*,delay_ = 1 − *w*_*ij*,direct_. The Boltzmann temperature is lowered when an output neuron spikes. If the Boltzmann temperature has reached its minimal value, the maximum of both weights converges to 1 and the minimum to 0.

For each layer, the weight setting has to be learned. The learning of the weights is a time evolving process. Weights of the first layer settle first and converge to their 0 or 1 end state. After the weights in the first layer have settled, the weights of the second layer begin to settle, consecutively the weights of the subsequent layers settle, until at last the weights of the last layer settle. Subsequent learning in each layer depends on the preconditioned setting of the weights in the previous layers. Learning finishes when all weights converged to their final states 0 or 1.

In summary, a neural net with velocity tuned cortical columns self-learns to detect bars of different slopes and sinusoids of different frequencies depending on the applied training set. Self-learning has been examined for different sizes of the neural net. The neural net executes a coordinate transform which maps the spatiotemporal input patterns through cortical columns and microcircuits to a feature vector. The solution found by the neural net is compared to mathematically derived solutions which are computed by Hough transform space-time equations for straight lines and sinusoids in a numerical grid. The authors drew the conclusion that the weight settings are either analytically derivable by the Hough transform equations or are self-learned by the neural net by pattern induced learning.

Based on its plausibility, the neuronal network model serves as a partial system model for functional aspects and dynamic properties of real neuron ensembles.

The signal propagation of temporary ordered sequences through the net incorporates synfire chains [39]. Its fine-tuned signal flow makes it a compelling model for cascaded firing and stimulus triggered signal propagation [40]. It strengthens the argument that spike-phase coding boosts and stabilizes information carried by spatial and temporal spike patterns [41]. The signal wave-front propagation is related to visual map and receptive field formation by signal wave induction [42].

A Boltzmann temperature has been introduced in the model so that the synapses converge smoothly to their final gating on or blocking off state which is consolidated by modeling synapses as binary open and closed gates [43]. Through this on/off switching to the minimal or maximal conductance an *in vivo* network flips from one state to another [44].

Unsupervised Hebbian learning has been assumed in the model which is in concordance with STDP [45]. In STDP, a synaptic link is potentiated or depressed where the positive or negative weight change depends on the relative time interval between the firing sequence of postsynaptic and presynaptic neurons. In contrast, the weights are strengthened or decreased by a fixed increment in the model here [46]. With a resource-dependent STDP variation complex temporal patterns can be learned [47]. These discrepancies and influences in weight which change according to applied learning rules should be investigated further by alternatively extending the model to include STDP learning rules. We have, for example, demonstrated that STDP learning behavior can be realized in a biohybrid synapse built as a memristor [48]. It is feasible to implement the model in neuromorphic hardware as in parallel memristor bridge synapse-based neural networks, as in an evolving spiking neural network with temporal spike learning, or as a model with implemented dendritic delays [49–51]. It can serve as a model to extract temporally correlated features with STDP from dynamic vision sensors [52]. Referential structures from videos can be partially encoded as examples demonstrating time encoding machines [53]. It can therefore serve as a model for the episodic nature of spike trains and for the relative spike time coding and STDP-based orientation selectivity in the early visual system [54, 55].

This model together with its foundation in basic neurobiological assumptions will serve as a subject in information processing and be a guide for system identification in further *in vivo* and *in vitro* experiments.

## 5. Novel *In Vitro* Protocol of the Hubel-Wiesel Experiment with 3D MEAs

The Hubel-Wiesel experiment using a new protocol presented here is planned to be revisited *in vitro* with stem cell based cocultured neuron-glia networks (NGN) and topologically selected stimulation electrodes from novel 3D MEAs.

The assumption is that receptive field areas will functionally assemble by synaptogenesis due to pattern induced stimulation in the NGN [56]. This would solve the pattern recognition task by having the *in vivo* environment essentially deliver the input stimuli mapped through cortical columns and microcircuits to the feature neurons.

The NGN is not developed according to a brain-like protocol nor does it resemble an explant neuron ensemble in its macro- and microstructure [57, 58]. Often self-spiking occurs after a few days and the network connects itself to partial microcircuits [59]. As there is no additive brain derived neurotrophic factor or other external signaling chemicals, the NGN will self-assemble and persist in its arrested self-spiking functionality if no pattern induced stimulation is applied and only partially dysfunctional local microcircuits will evolve [60, 61].

Astrocytes and neurons combine in a homeostatic relationship to create the so-called tripartite or tetrapartite synapse [62, 63]. Astrocytes therefore play a crucial role in the retrograde signaling cascade enabling timing long-term depression (tLTD) which is a prerequisite for changing the synaptic link strength in experiments [64]. Synaptic connections strengthen or weaken by modifying the presynaptic glutamate release probability by activating presynaptic located ionotropic receptors [65].

Several *in vitro* experiments which have been conducted by various researchers with paired-pulse single electrode or paired-pulse electrodes stimulation partially support the feasibility that an evolving NGN can reproduce the model.

Activity can be induced by electrode stimulation of a single electrode in near-distant neurons [66]. The activation can be traced back directly to the conducting axonal branches of the stimulation neuron in the vicinity of the place of innervation by the electrode and therefore local connections of neurons can build up activation chains of consecutive firing sequences.

Repetitive paired-pulse stimulation of a single electrode for brief periods induces persistent strengthening or weakening of specific polysynaptic pathways depending on the inter-pulse interval [67]. Correlated pre- and postsynaptic excitation at distant synapses is due to different transmission delays along separate pathways. Through such a delay-line mechanism, temporal information coded in the timing of individual spikes can be converted into and stored as spatially distributed patterns of persistent synaptic modifications. As a result, the evolution of tiny timing microcircuits might be reflected in the experiment. With paired-pulse stimulation with distant remote electrodes, cultured cortical networks are able to learn by shuffling the timing impulse responses of the neurons relative to the applied inter-pulse intervals [68]. This supports the assumption that time can be precisely stored in time ladders. In a ring-shaped cultured cortical network, a prolonged activity can be triggered by electrode stimulation, in which a sequence of neurons fires cyclically in a ringing mode following a single stimulus [69]. This supports the model assumptions of concatenated neuron sequences.

**Figure 4:**
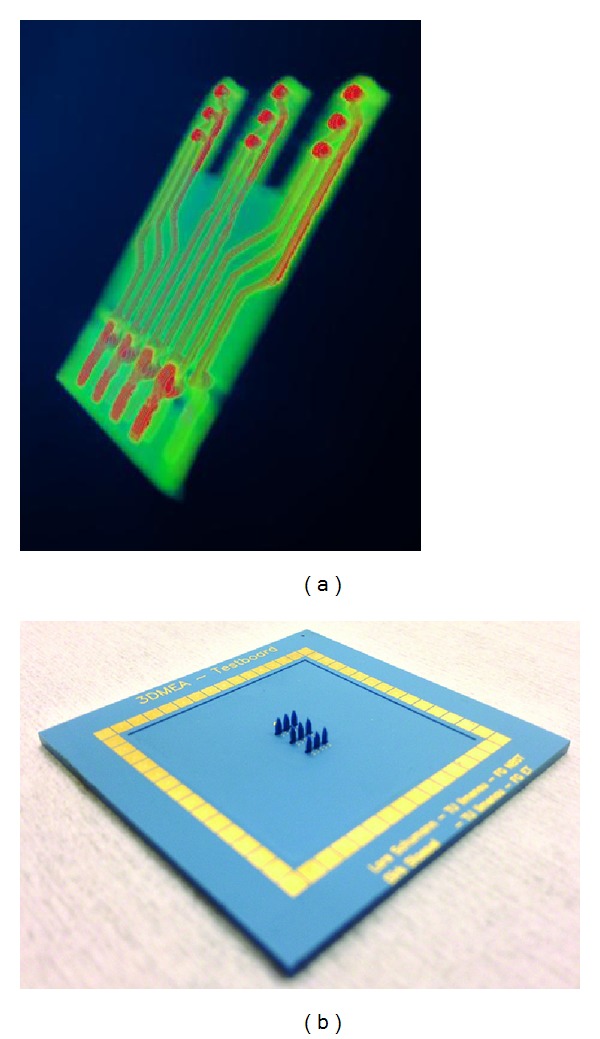
Micro-CT image of a microtower with integrated electrodes which forms a 2D MEA (a) and which forms a 3D MEA (b) when placed in series with a baseplate compatible with commercial amplifier systems.

Cultured cortical networks *in vitro* from explant cultures, dissociated cell cultures, and stem cell derived neuron ensembles can be stimulated in a controlled fashion by electrode stimulators from different vendors, such as Multi Channel Systems and Plexon [70]. Several stimulation electrodes of a MEA can be selected and stimulation sequences can be streamed to the electrodes in parallel, with front-end electronics operated by computer assisted control and monitoring modes. The NGN will be stimulated by applying specific stimulation sequences to the electrodes [71]. The stimuli will be provided to a 3D NGN environment using 3D MEAs created at the Technische Universität Ilmenau, shown in (Figure 4) [72, 73].

**Figure 5:**
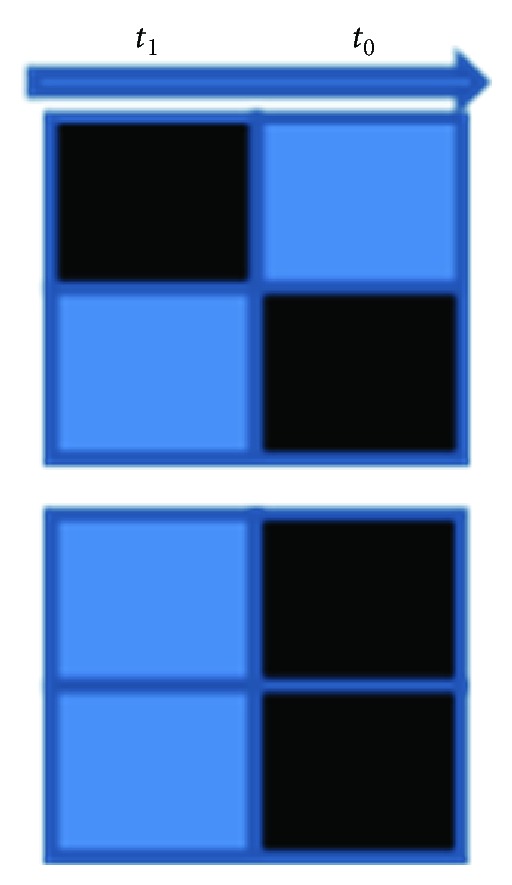
Two spatiotemporal stimulation sequences to be provided by the microelectrodes of the 3D MEA.

Contrary to *in vivo* experiments, no visual cues such as physical bars presented to the retina will be necessary. Instead, the bars will be presented as parallel aligned activated sets of electrodes in a pixel-wise fashion (Figure 5). Spatiotemporal pattern sequence activation is adopted. The sequences are produced like playing a piano with both fingers. At time *t*_0_, electrode 2 is activated. At consecutive time *t*_1_, electrode 1 is activated. The NGN sees a diagonal bar as a spatiotemporal sequence. It is paired-pulse stimulation in a predefined time lag interval over two adjacent electrodes. For the second training pattern—a vertical bar—electrode 1 and electrode 2 are activated in parallel (Figure 5).

## 6. Learning and Long-Term Structuring of the Neuron-Glia Net

The patterns will be presented by the 3D MEAs over long stimulation times to the cultured cortical network. Neurons in the vicinity of the electrodes will be activated consecutively in time and the computations will remain local by limited axonal and dendritic outspread. Some of the questions will be as follows. Are input stimuli mediated through cortical columns and microcircuits to feature neurons? Are the networks able to discriminate between the presented patterns? Are the feature detector neurons arranged in an ordered fashion according to ascending feature sets? And are all intermediate neurons intrinsically localized as part of the cortical microcircuit in order to realize an identification of the system?

The cortical column model is adopted for system inspection and is essentially modeling aspects associated with the evolving NGN. This is a numerical model and as such has some unitary parameters which are not related to physical entities, like absolute timing in measurable quantities. Time is unitary and specified as a clock step with no time associated units in the model. In the experiment for STDP learning, the time interval will be set to the millisecond range according to literature values. The inter-pulse interval will be set in the stimulator. With the help of the experiment, we plan to fine-tune the model and parameters of the physical NGN. In variations of the experiment, the control parameters will be tuned to see where the optimal range and set points of various controllable parameters are. These parameters and points include stimulus length, STDP timing intervals, repetitive pattern burst modes, number of training cycles, and day of stimulus activation. A crucial role is played by the adaption of the weights to their minimal blocking values or maximal conducting values, respectively.

Thus far, our computer simulation of the cortical column model requires the weight incrementing learning curve value *α* to be set to a very low value. This requires that the training patterns be applied several hundred thousand times. However, the parameter *α* can be determined in these experiments by counting the number of training cycles until a steady truth table configuration has been reached. The minimal constituting elements of the cortical columns microcircuit are two input neurons, four delay neurons, and two output neurons to detect the patterns in Figure 5 which are covered in the recording MEA field. The truth table for the cortical microcircuit is as follows: if input neuron 1 and input neuron 2 are on at time *t*_0_, then output neuron 1 is on; if input neuron 2 is on at *t*_0_ and input neuron 1 is on at time *t*_1_, then output neuron 2 is on.

An early indicator for the success of the experiment is that over many stimuli test patterns some of the recording electrodes will have a high output value relative to the trigger time of the applied stimulus. In a subsequent observation, the first two delay neurons in the cortical column can be disregarded by directly connecting the axonal branches to the dendrites of output neuron 1, which detects the coincident firing of the two input neurons. As a result, these neurons will fire. The key learning is experienced by the two delay neurons in cortical column 2. Over the sustained training period, it is necessary and sufficient that the synaptic branch tends towards the delaying neuron 2 and the direct branch connectivity is cut off. In a coevolutionary manner, the upper signaling path should establish a direct axonal connection to the dendrite of output neuron 2, so that neuron 2 can sense the synchronous coincidence of the upper and lower signaling path.

## 7. Risk Assessment

The risks of the experiment are clearly identified. The cortical microcircuit elements are very few and it is not *a priori* assured that the activities of the constituting elements are covered by electrodes in the vicinity. The system could be underdetermined or overdetermined resulting from hidden neurons not covered in the system playing a crucial role in the microcircuit. The model adopted could be wrong with nature implementing its logic circuitry in another fashion. To investigate the string-like trajectory information processing the input layer size will be enlarged as soon as more stimulation electrodes become available for the experiment.

## 8. Conclusions

A thorough description on different levels of abstractions has been given to reproduce the Hubel-Wiesel experiment *in vitro*.

The Hubel-Wiesel experiment has been resumed and the contributions of several authors were listed, which indicate some plausibility for the mathematical Hough transform as substrate of information processing in biological maps for orientation selectivity. Several computational Hough models at neural level have been compared and one model has been selected as a guide for further experiment. The proposed *in vitro* experiments will be guided as presented in the paper here. The main goals are to see if our model assumptions can be verified by experiment and if an indication of evidence for information processing can be definitely given in a cortical column microcircuit.
